# Review: Failure of current digoxin monitoring for toxicity: new monitoring recommendations to maintain therapeutic levels for efficacy

**DOI:** 10.3389/fcvm.2023.1179892

**Published:** 2023-07-03

**Authors:** Sridhar Rao Gona, Joel Rosenberg, Ria C. Fyffe-Freil, Janet M. Kozakiewicz, Mary E. Money

**Affiliations:** ^1^ Department of Pharmacy, Meritus Medical Center, Hagerstown, MD, United States; ^2^ MedStar Cardiology Associates, Washington, DC, United States; ^3^ Division of Clinical Biochemistry & Immunology, Department of Laboratory Medicine and Pathology, Mayo Clinic, Rochester, MN, United States; ^4^ Independent Healthcare Consultant, Trumbull, CT, United States; ^5^ Department of Medicine, University of Maryland School of Medicine, Baltimore, MD, United States; ^6^ Department of Medicine, Meritus Medical Center, Hagerstown, MD, United States

**Keywords:** digoxin, digoxin toxicity, therapeutic level, narrow therapeutic index and critical dose drugs, rapid atrial fibrillation/flutter, digoxin morbidity, digoxin monitoring recommendations, patient quality and safety

## Abstract

The current recommendations for monitoring digoxin, a narrow therapeutic index drug, are limited to confirming medication use or investigating suspicion of toxicity and fail our oath to do no harm. Numerous meta-analyses evaluating digoxin use consistently recommend frequent monitoring to maintain the level of 0.5 to ≤1.0 ng/ml because higher levels lead to increased morbidity and mortality without benefit. Data from the United States National Poison Control Center (2012–2020) show annual deaths due to digoxin of 18–36 compared to lithium's 1–7, and warfarin's 0–2 respectively. The latter drugs also have narrow therapeutic indexes like digoxin yet are more carefully monitored. Recognition of digoxin toxicity is impaired as levels are not being routinely checked after medications are added to a patient's regimen. In addition, providers may be using ranges to guide treatment that are no longer appropriate. It is imperative that monitoring guidelines and laboratory therapeutic levels are revised to reduce morbidity and mortality due to digoxin. In this review, we provide a comprehensive literature review of digoxin monitoring guidelines, digoxin toxicity, and evidence to support revising the ranges for serum digoxin monitoring.

## 1. Introduction

Digoxin has been used for cardiovascular diseases for centuries and patients have been followed clinically to observe for toxicity (1). Monitoring guidelines for digoxin were not established until a radioimmunoassay was developed in the late 1960's (2). Those guidelines, however, recommended monitoring only to confirm compliance or toxicity (3). Close monitoring after the addition of another medication was recommended and not required (4). In addition, routine monitoring of digoxin, for use in atrial fibrillation, was discouraged since it was believed that higher levels were necessary for rate control (5). By the 1970's the therapeutic range for digoxin was established to be 0.8–2.0 ng/ml (6). The first retrospective review of the risk/benefit of digoxin was by Rathore, et al. in 2003 (7). The authors demonstrated increased mortality when digoxin levels were above 1.2 ng/ml and with minimal effectiveness. This led to subsequent studies and formal recommendations for the therapeutic level of digoxin to be lowered. Unfortunately, these new levels have not been fully adopted in the medical references nor by testing laboratories. Because the symptoms of digoxin toxicity are heterogeneous and nonspecific, toxicity is often underrecognized by providers and the death rate from digoxin, when compared to other drugs with narrow therapeutic indexes, is excessive.

### 1.1. Case report and subsequent retrospective chart review

Regrettably, the guideline landscape for digoxin serum monitoring creates confusion among providers and anxiety and frustration among patients and their families. We will share a case study of unrecognized digoxin toxicity at Meritus Medical Center, a 300-bed community teaching hospital in Maryland. It is this case that prompted a retrospective chart review (RCR) of patients admitted with toxic serum digoxin levels >2 ng/ml and to evaluate the relationship of the addition of new medications, added to the patient's medication regimen, as contributing factor to toxicity. The results of this RCR have also prompted changes to this institution's digoxin monitoring guidelines.

This is the case of an 80-year-old female who had been on digoxin for 6 months for rate control for atrial fibrillation/flutter. Subsequent to starting digoxin she was started on numerous other medications and developed generalized weakness, confusion, nausea, vomiting, and change in vision. Over the course of these 6 months, she was seen by multiple providers who failed to recognize the symptoms of digoxin toxicity, and who failed to check a serum digoxin level after the initiation of numerous medications. When a serum digoxin level was measured, the level was 3.31 ng/ml (Case report in Supplementary Material).

**Figure 1:**
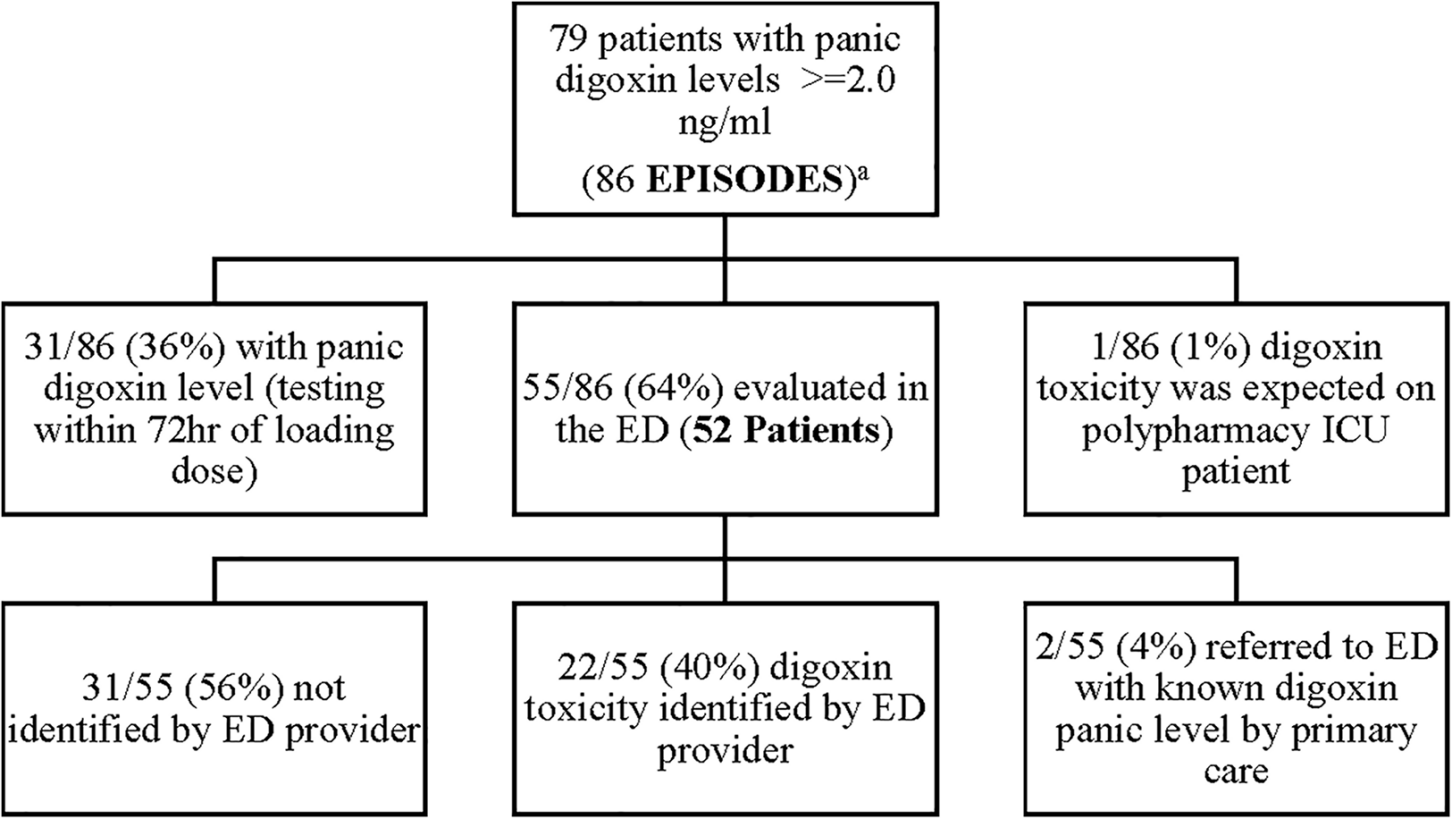
Study analysis.

A retrospective, Electronic Health Record (EHR), chart review was performed at Meritus Medical Center from, April 1st, 2019, to April 1st, 2022, on all patients admitted with a toxic serum digoxin level. Patients were then evaluated to see if the toxicity was due to a new medication added to the patient's regimen. At the time of the evaluation, the established therapeutic range for digoxin at Meritus Medical Center was 0.8–2.0 ng/ml. During this time period 86 episodes of serum digoxin levels (panic levels), greater than or equal to 2.0 ng/ml, were identified (Figure 1). Of these 86 episodes, 31/86 (36%), had toxic digoxin levels with testing performed within 72 h of the intravenous administration of digoxin for control of rapid atrial fibrillation or flutter. Only one digoxin level ≥2.0 ng/ml per admission was included in the analysis. The remaining 55 episodes (52 patients) were all evaluated in the Emergency Department (ED) (Tables 1, 2). Each of these cases was carefully reviewed to collect the following information: demographic data; reason for and time when the level was drawn; original date patient was given the prescription or was documented to be taking it; symptoms or physical findings suggestive of toxicity recorded in the EHR on admission; potential drugs prescribed contributing to the elevated level; date and value of the most recent digoxin level prior to admission; provider specialty who initially prescribed digoxin; number of concomitant medications; and patients current health status, as of the study date. If available, the time interval between the toxic level and death was also documented Tables 1, 2.

**Table 1:** Characteristics of patients with digoxin toxicity admitted through emergency department.

Patient population characteristics *n* = 52	
Gender	
Females	38
Males	14
Mean ± SD age	77.59 ± 9.16 years
Mean ± SD weight (Kg)	88.20 ± 30.07
Mean ± SD serum digoxin level	2.81 ± 0.70 n
Patient status at time of review	
Alive	19
Deceased	33
Race	
Black or African American	2
Caucasian	49
Native Hawaiian/Other Pacific Islander	1
Ethnicity	
Non-Hispanic	52
Initial digoxin prescriber	
Cardiologist	27
Primary Care	2
Hospitalist	1
Unknown	22
Interval between the last digoxin level prior to admission (months)^a^	
Less than 3	22
>3 but ≤6	3
>6 but ≤12	1
>12 but ≤18	1
>18	1
Unknown	24
Number of other prescription medications taken by each patient	
<5 Rx Meds	8
5–10 Rx Meds	6
>10 Rx Meds	38

^a^ Refer to Table 2 for values of prior digoxin level if obtained.

**Table 2:** Serum digoxin level value prior to admission when obtained.

Serum digoxin level	No. of patients
<0.2 ng/ml	2
≥0.2 to ≤0.5 ng/ml	4^a^
≥0.5 to ≤0.9 ng/ml	4
≥0.9 to ≤1.2 ng/ml	7
≥1.2 to ≤2.0 ng/ml	13
≥2.0 ng/ml	2
Unknown	23

^a^ Four patients had the following digoxin values prior to admission: 0.207, 0.221, 0.258, 0.483 ng/ml.

One out of 86 (1%) was anticipated as the patient (who was on digoxin at the time of admission) was in ICU, numerous medications were initiated, and a serum digoxin level was monitored daily for six consecutive days until the level became toxic. There were 2/55 (4%) patients referred to the ED with a known toxic level and 22/55 (40%) of episodes were identified by the ED provider. Disappointedly, 31/55 (56%) were not recognized by the ED provider, leading to a delayed recognition of digoxin toxicity.

In 58% (32/55) of cases, a new medication was started prior to the patient's admission to the ED. The drug classes contributing to the digoxin toxicity included antibiotics, antidiuretics, proton pump inhibitors, and antiarrhythmics. In each case, there was no evidence, in the EHR, that a serum digoxin level had been obtained. In 27% (15/55) of patients, digoxin had been previously started in the hospital for control of rapid atrial fibrillation. No serum digoxin level was found until the patient was readmitted with a toxic level. This practice, however, was in line with the recommendation that checking serum digoxin levels when used for atrial fibrillation is not indicated.

**Figure 2:**
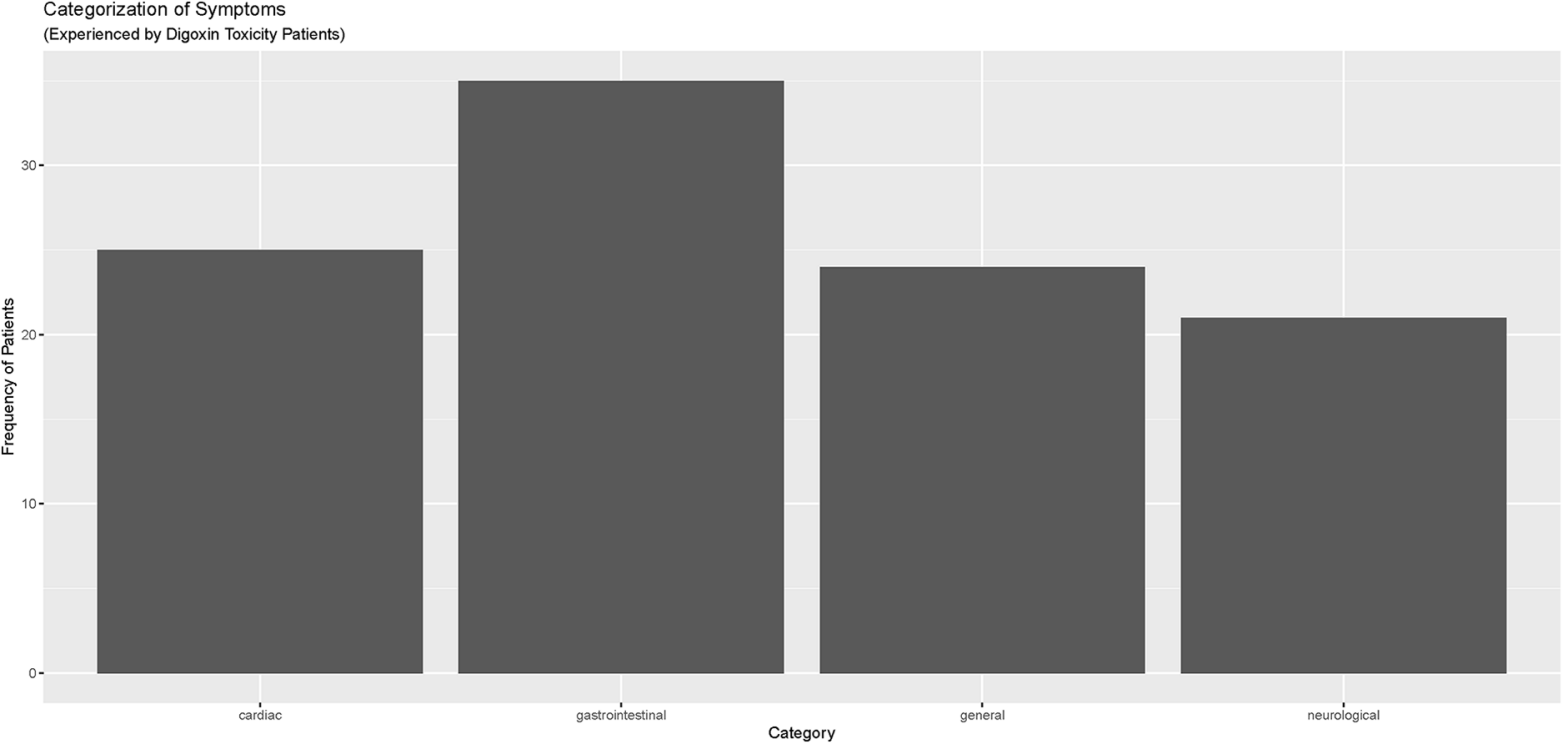
Categorization of symptoms reported by digoxin toxicity patients in meritus medical center cohort (April 1, 2019–April 1, 2022).

**Figure 3:**
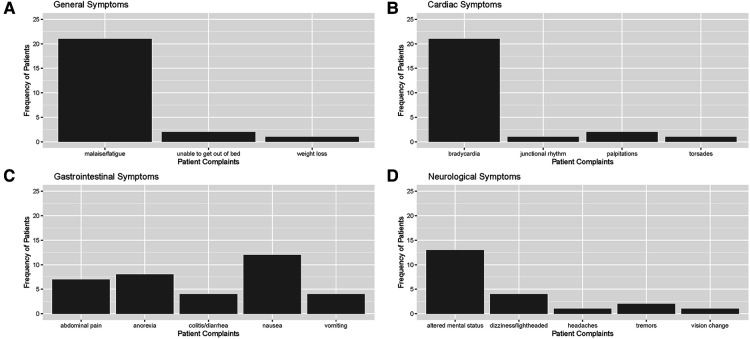
Distribution of documented symptoms among patients with toxic digoxin levels in ED or admitted to meritus medical center from April 1, 2019, to April 1, 2022 in the following categories: (**A**) general/constitutional, (**B**) cardiovascular, (**C**) gastrointestinal, and (**D**) neurological (including visual changes).

Electronic Health Records (EHRs) were carefully reviewed to identify documented symptoms or physical findings that suggested digoxin toxicity (Figures 2, 3). Only one patient, referred directly to the ED from a nursing home for suspected digoxin toxicity was documented to have no symptoms. The presence or absence of documented symptoms may not accurately reflect whether the patient experienced digoxin toxicity. This may be due, in part, to the nonspecific clinical manifestations of digoxin toxicity that may mimic that of other chronic diseases. In this review, documented general symptoms of malaise/fatigue occurred with equal frequency as bradycardia (Figures 3A–D). It is important to note that cardiac arrhythmias occurred in <50% of this cohort of patients presenting to the emergency department and whose digoxin level was >2.0 ng/ml.

Limitations of this study include the following: only data from the EHRs of Meritus Medical Center hospital and its affiliated outpatient centers was assessed; digoxin levels obtained at an outside, unaffiliated, laboratory were not included in this review; review of any changes in renal function was not performed; and any acute illness present prior to admission, that may have contributed to the digoxin serum level elevation, was not reviewed.

### 1.2. Difficulty recognizing digoxin toxicity symptoms

The common complaints suggestive of digoxin toxicity, are heterogeneous and commonly present, specifically in the geriatric population who have multiple comorbidities (Table 3). These can easily be mistaken by providers of care with symptoms of the normal aging process (11, 12). The toxic effects of *digitalis* (the plant family from which digoxin was isolated) have been recognized since William Withering's original manuscript. His description included “sickness, vomiting, purging, giddiness, confused vision, objects appearing green and yellow; increased secretion of urine, with frequent motions to part with it; slow pulse, even as slow as 35 in a minute, cold sweats, convulsions, syncope, and death” (1). Patients often present to their primary care providers with a variety of vague symptoms that could be attributed to any number of comorbid health concerns. Lapses in oversight may occur when a specialist, such as a cardiologist, orders the digoxin and the primary care provider wrongfully assumes that the specialist is monitoring the levels. Also, they may not be aware that a serum digoxin level should be obtained after starting a patient on a new medication. Likewise, cardiologists are frequently not aware when a patient is prescribed a new medication by another provider and hence serum digoxin levels are not obtained.

**Table 3:** Reported symptoms associated with digoxin toxicity (8–10).

Organ system	Potential toxicity manifestations
General	Fatigue, malaise, insomnia, loss of interest, depression, anxiety, restlessness, weakness,
Gastrointestinal	Anorexia, nausea, vomiting, diarrhea, abdominal pain, constipation, Intestinal ischemia
Cardiac	Worsening of heart failure, Atrial arrhythmias, A-V block, Ventricular arrhythmias, A-V nodal arrhythmias.
Neurological complaints	Headache, confusion, altered mental status, vertigo, trigeminal neuralgia and other neuralgias, convulsions, paresthesias, delirium, psychosis, hallucinations
Visual complaints	Blurring, color changes: particularly green or yellow hues with possible halos, rarely: scotomas, micropsia, macropsia, amblyopias
Other rare manifestations	Urticaria, eosinophilia, thrombocytopenia, gynecomastia, macular popular rash, hyperkalemia

As a result, ED visits due to digoxin toxicity and subsequent hospitalization continues to pose a major problem for hospitals and health care systems. Data from the National Electronic Injury Surveillance System-Cooperative Adverse Drug Event Surveillance Project and the National Ambulatory and Hospital Ambulatory Medical Case Surveys (2005–2010) revealed an estimated 5,156 visits to the ED annually for digoxin toxicity in the United States with more than 75% resulting in hospitalizations (13). Patients greater than 85 years of age were twice as likely to present to the ED with toxic digoxin levels than those 40–84 years of age and represented 3.3% of ED visits and 5.9% of hospitalizations related to adverse drug events. Although the number of prescriptions for digoxin had decreased during this time, the number of ED visits remained constant. Budnitz et al, using the same data source for the years 2007–2009, concluded that among the commonly implicated medications for adverse events requiring hospitalization, digoxin was the third highest at 5 hospitalizations per 10,000 medication visits (14). In 2016, Limon et al. retrospectively evaluated ED patient visits from January 2010 to July 2011 who had digoxin levels ≥1.2 ng/ml for symptoms of digoxin toxicity (15). Digoxin levels were measured in 851 patients, with 139 (16%) showing a level ≥1.2 ng/ml; of these patients 41 (29.5%) had symptoms of toxicity, 2 (1.4%) were excluded, and 96 (69%) were not intoxicated. Although cardiac symptoms were the most common diagnosis in the intoxicated group, 26.8% had no EKG changes. Other symptoms included nausea and vomiting (35%), abdominal pain (27.5%), and neurological changes (5.1%) (15). Finally, Peters et al, using a serum digoxin level of >2.0 ng/ml to indicate toxicity, did a retrospective analysis of EHRs at Duke University Health System from 2000 to 2020 (16). A total of 779 encounters were evaluated for digoxin toxicity over this 20-year period with 93% having elevated serum digoxin concentrations defined as >2.0 ng/ml. The author also noted that only 3% of the cohort had an ICD billing code for digoxin toxicity. Since many RCRs rely heavily on ICD billing codes to identify adverse medication events, the potential exists to underestimate the prevalence of digoxin toxicity.

### 1.3. Risk of digoxin toxicity and polypharmacy

Recommendations made by Marcus (17) in *Pharmacokinetic Interactions Between Digoxin and Other Drugs* did not have the same impact as that of Selzer in 1985. Marcus strongly advised physicians to “maintain constant vigilance whenever medications are added to or withdrawn from a therapeutic regimen that includes digoxin”. Currently UpToDate, an evidence based clinical decision resource, notes that numerous medications may impact the digoxin level and “serum digoxin levels higher than 1.0 ng/ml (1.3 nmol/L) should be avoided to reduce the risk of toxicity”, and “Given the relatively narrow therapeutic window of digoxin and substantial overlap between therapeutic and toxic levels, patients taking digoxin require monitoring of the serum digoxin concentration” (18). It is clear that more diligent monitoring is needed due in part to the aging population, their numerous comorbidities and medications that accompany this advanced age. A 2015 review of 367 patients in New South Wales, Australia with atrial fibrillation (mean age 77.8 years) found that 94.8% used >5 medications and over 50% used ≥10 medications. In this published cohort, 30.2% were taking digoxin of which only 21.6% had heart failure (19). In 2011 Currie noted that “In the elderly, special consideration and dispensation needs to be applied for digoxin's narrow therapeutic index, co-morbid diseases, polypharmacy and altered pharmacokinetics to minimize toxicity and sub-optimal therapy” (20).

The lack of attention to serum digoxin monitoring is often driven by several mitigating factors. While recommendations regarding therapeutic drug monitoring for narrow therapeutic index drugs have been updated by the United States Food and Drug Administration (FDA), the recommendations for digoxin have remained unchanged. Mistaken concerns regarding insurance coverage for serum digoxin testing although speculative, is also a strong deterrent. A recent review of The Centers for Medicare Services National Coverage Determination states that serum digoxin determination is covered when “additional medications are added that could affect the digoxin level” (21). Providers of care now rely heavily on clinical decision support within the various EHRs to provide drug-drug interaction (DDI) alerts. Depending on the sensitivity set within the system, however, it may not include all the drugs that adversely impact digoxin levels. To minimize “alert fatigue” many EHRs only trigger DDI alerts for the most “severe” interactions (22). Also, there are major discrepancies among references as to what drugs adversely impact digoxin levels. Table 4 provides a comparison of the number of medications listed as potentially affecting the digoxin therapeutic level by source.

**Table 4:** Number of drugs listed in each reference that could potentially interact with digoxin.

Source (ref)	Number of drugs potentially reacting with digoxin
Lexicomp (18, 23)	139
FDA (24)	61
Drugs.com (25)	416
Epocrates.com (26)	346

This variability among data sources, created to help reduce patient harm and relieve pressure on staff, have created cause for alarm. Current constraints on provider time prevent them from exploring multiple drug information sources. Moreover, this variability compels providers of care to rely heavily on pharmacists to carry the burden. Unfortunately, retail pharmacies often do not have all of the patient medication history required to perform a complete DDI screening. Patients often use multiple pharmacies for their prescription needs or they may be an unreliable source of medication information. This makes it essential to have an accurate drug interaction screening completed, in real time, as the order/prescription.

### 1.4. The digoxin therapeutic range and reported risk of mortality

Although plants in the foxglove (or *digitalis*) family have likely been used as medicinal therapy for centuries, its flowers have been long known to sometimes hurt and sometimes heal. In the 18th century, William Withering's manuscript regarding its effect on 163 patients treated by him resulted in the recognition of the benefit for patients with “dropsy”, later identified as heart failure (1). Two of the most common foxgloves, *Digitalis purpurea* and *Digitalis lanata*, contain active components that are classified as cardiac glycosides and are indicated for treatment of mild to moderate heart failure, increasing myocardial contractility in pediatric patients with heart failure, and control of ventricular rate with chronic atrial fibrillation or flutter. Digitalis works by inhibiting the cellular membrane sodium-potassium adenosine triphosphatase and improves cardiac contractility. The first branded digitalis (digoxin), Lanoxin tablets® for oral use, was approved by the FDA in 1954 (24). Originally used extensively to treat congestive heart failure, its use has fallen out of favor due to novel entities with less potential for toxicity. Currently, its use is restricted primary for rate control when faced with hypotension from concomitant medication such as β-blockers or calcium channel blockers (27).

Digoxin has a narrow therapeutic index and is associated with a high incidence of toxicity; this has been well-established for decades. It is rapidly absorbed, reaching a peak serum concentration in 1–3 h, and steady state in 7–12 days in patients with normal renal function. Toxicity can often develop after the addition of a new medication that directly affects digoxin metabolism or renal clearance, particularly antibiotics. Bacteria in the gut metabolize digoxin and form an inactive reduction product. Consequently, co-administration of certain antibiotics may raise the serum level (10). Although the FDA labeling for digoxin has strong recommendations for monitoring when certain medications are added, this is not being done routinely as it is with other narrow therapeutic index drugs, like warfarin or lithium due to their potential for toxicity (Table 5).

**Table 5:** Comparison of deaths with lithium, warfarin, and digoxin as reported to the US poison Center's national poison control from 2012 to 2020.

Year (ref)	Lithium			Warfarin			Cardiac glycosides (digoxin)		
	Lithium + Other drugs	Single drug	Deaths	Warfarin + Other drugs	Single drug	Deaths	With other drugs	single drug	Deaths
2012 (28)	6,663	3,443	2	3,777	2,022	0	2,525	1,652	18
2013 (29)	6,610	3,488	5	3,601	1,864	0	2,342	1,468	25
2014 (30)	6,850	3,597	7	3,402	1,766	0	2,230	1,432	38
2015 (31)	7,143	3,825	1	3,247	1,660	1	1,916	1,253	18
2016 (32)	6,901	3,715	2	3,025	1,532	0	1,905	1,252	26
2017 (33)	7,222	3,827	2	2,604	1,251	2	1,851	1,234	25
2018 (34)	7,055	3,865	3	2,342	1,136	0	1,689	1,143	23
2019 (35)	7,748	3,033	2	2,227	1,066	0	1,623	1,138	27
2020 (36)	6,276	3,420	4	1,681	799	1	1,498	1,043	28

This lack of monitoring may stem from Selzer's 1985 manuscript, “*Role of Serum Digoxin Assay in Patient Management*” in which he stated: “Buildup of digoxin levels to what is generally considered a toxic range is frequently necessary to adequately control ventricular rate”. He goes on to say, “Clinical criteria for possible toxicity, rather than serum assay, are used to set the limit of digitalis in patients whose ventricular rate cannot be controlled by digitalis alone.” He then concluded that the serum digoxin level “can be used as a check of patient compliance, particularly when a patient is known to have had adequate levels in the past”, and “Digoxin levels above 2 ng/ml do not prove digitalis toxicity. They merely indicate that toxicity is possible” (4). These statements appear to support the concept that awareness of routine serum digoxin levels is not necessary and should only be obtained if toxicity is suspected.

Current monitoring guidelines in the web-based practice resource UpToDate states that “Monitoring the serum digoxin concentration is most important when digoxin is used in the treatment of heart failure with systolic dysfunction, whereas levels are only checked when used in patients with atrial fibrillation if toxicity is suspected.” It goes on to say “When digoxin is used strictly for ventricular rate control in atrial fibrillation, serum digoxin levels should be monitored periodically, although the drug concentration often does not correlate with ventricular rate control and is used more as a guide to toxicity than to therapy” (18).

The establishment of toxic level for digoxin at >2.0 ng/ml was based on studies done in the late 1960's and presented at the 40th Scientific Sessions, American Heart Association in Dallas, Texas. The conclusions were based on adult patients who had been on a maintenance dose of digoxin for 5 or more days and admitted to Massachusetts General Hospital from September 1968 through December 1969. They reported that 131 patients were found not to have digoxin toxicity, 48 had cardiac rhythm changes compatible with toxicity, and 48 were diagnosed with equivocal or vague signs of digoxin toxicity. Patients were carefully interviewed for symptoms of digoxin toxicity and EKGs were interpreted by independent cardiologists not involved in the study. Serum digoxin levels were measured using a radioimmunoassay method (37). Findings were as follows: “Despite comparable mean daily digoxin dosages, digoxin intoxicated patients had a mean serum digoxin concentration of 3.7 ± 1.0 (_SD_) ng/ml, while nontoxic patients had a mean level of 1.4 ± 0.7 ng/ml (*P *< 0.001). Ninety percent of patients without evidence of toxicity had serum digoxin concentrations of 2.0 ng/ml or less, while 87% of the toxic group had levels above 2.0; the range of over-lap between the two groups extended from 1.6–3.0 ng/ml” (3).

By 1974, the therapeutic range of digoxin was firmly established as 0.8–2.0 ng/ml and levels <0.8 ng/ml were recognized as subtherapeutic, based on a study by Carruthers. This prospective study evaluated 101 patients admitted to the Belfast City Hospital in Northern Ireland ED on chronic digoxin therapy (6). The authors used limited criteria for toxicity which included the following: (1) nausea and/or vomiting that resolved when digoxin was discontinued without other etiologies, and (2) specific cardiac arrythmias. Based on this criterion, toxicity was present in 13/101 patients, (average age 70.9 + 9.0 years) with a mean plasma digoxin concentration of 2.76 ng/ml by radioimmunoassay.

In 1999, Cañas and colleagues published a review titled “*Evaluating the Appropriateness of Digoxin Level Monitoring*” (38). They set out to establish the appropriateness of random samples of serum digoxin levels in both inpatient and outpatient subjects. By augmenting previously published guidelines with expert opinion they used the following criteria to assess the appropriateness of digoxin level monitoring:

1) Subtherapeutic response due to: No improvement or worsening of heart failure or atrial fibrillation/flutter, suspected noncompliance, concomitant use of interacting medications, and suspected malabsorption.
2) Suspected toxicity due to: Appearance of arrhythmias potentially due to digoxin, or noncardiac signs of toxicity
3) High-risk patient with change in renal function, diuretic therapy, electrolyte abnormalities
4) Obtained 10 days after the patient was started on digoxin and had achieved a steady state
5) Obtained upon admission to the hospital if not done within the last 9 months and no evidence of toxicity
6) Obtained as an outpatient on a stable dose of digoxin after a 10-month interval.

The results revealed that only 16% of inpatient levels were appropriate, and many of the inappropriate values were due to levels being drawn before a steady state plasma concentration had been achieved. There were no recommendations regarding monitoring the serum levels after a new medication was started and the number of drug-drugs interactions was limited to those available in 1999 (38).

Important considerations from the Carruthers and Cañas studies, 25 years apart, were the acceptance that levels <0.8 or 0.9 ng/ml were “subtherapeutic”, and the mean age of patients taking digoxin was 70 and 68, respectively. Monitoring to maintain a therapeutic concentration was not included in either study.

Although digoxin is no longer considered a first line treatment for congestive heart failure (39), it is still being used for rate control in atrial fibrillation when hypotension develops due to attempts to increase the dose of other cardiac medications (40). With the aging population, atrial fibrillation is becoming a “global epidemic” (41). The number of patients with atrial fibrillation is expected to reach 12.1 million in 2030 in the United States (42). The Centers for Disease Control and Prevention (CDC) estimated that 9% of individuals aged 65 and older have atrial fibrillation. A recent retrospective study of 97 very elderly patients (>90 years of age) admitted to the emergency department over the last 5 years identified 40.2% had atrial fibrillation, and over half with rapid ventricular response (43). According to Clinical Drug Stats, there were 1,905,633 digoxin prescriptions in 2020, down from 6,475,250 prescriptions in 2013 (44).

**Figure 4:**
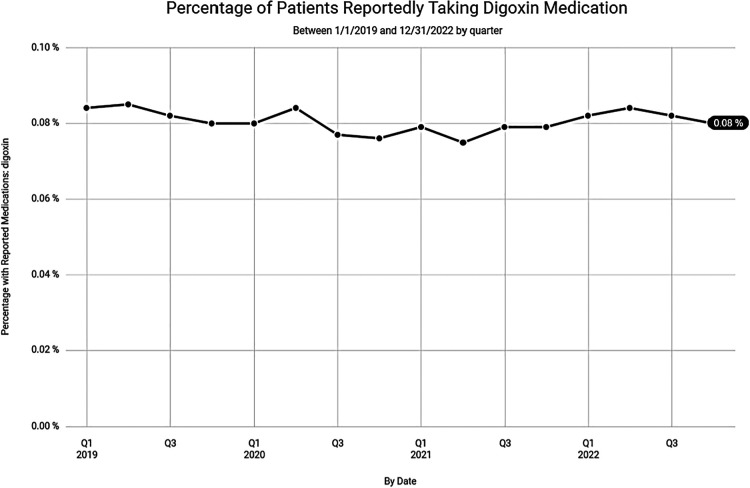
Percentage of patients reportedly taking digoxin over the last 3 years according to data from Epic Cosmos.

**Figure 5:**
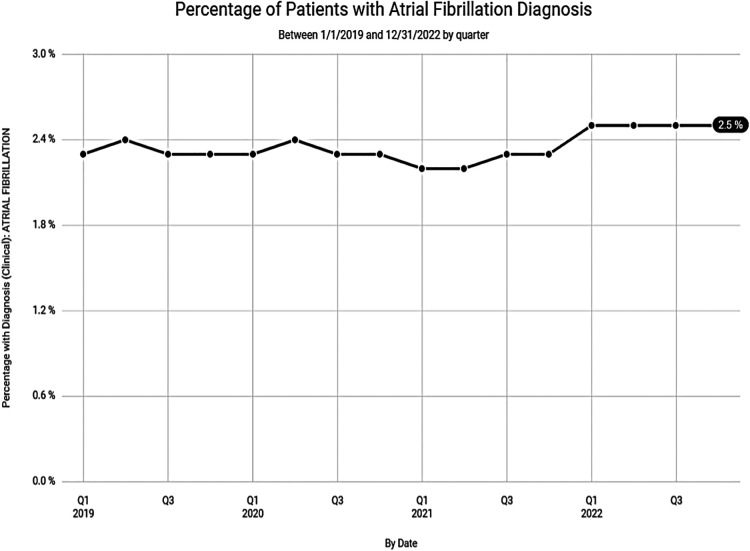
Percentage of patients with atrial fibrillation diagnosis over the last 3 years according to data from Epic Cosmos.

Despite the lower numbers of digoxin prescriptions noted in Clinical Drug Stats, recent data, mined from the EPIC EHR, representing 1,123 medical facilities, tells a much different story. Our data suggests that the steady decline in digoxin use, experienced prior to 2019, has waned with recent use remaining relatively steady (Figure 4). This may be related to the anticipated rise in prevalence and the increased incidence of atrial fibrillation found in our data among seniors (Figure 5).

In 2003, Rathore et al., evaluated the association of digoxin level and mortality in patients with heart failure by performing a *post hoc* analysis of the 1991–1995 DIG trial (45, 46). In the DIG trial, a double-blinded placebo trial, men with heart failure were randomized to either placebo (*n* = 2,639) or digoxin (*n* = 2,642) with the intent of achieving a serum digoxin concentration of 0.5–2.0. The study population, reviewed by Rathore nearly a decade later, consisted of men with heart failure randomized on placebo (*n* = 2,611) or digoxin (*n* = 1,171). Serum digoxin levels were obtained 1 month after initiation and 6 h after oral intake. The men were assigned to three groups with their serum digoxin levels as follows: 0.5–0.8 ng/ml (*n* = 572), 0.9–1.1 ng/ml (*n* = 322), ≥1.2 ng/ml (*n* = 277). The study identified that patients who had a level of 0.5–0.8 ng/ml had a lower mortality rate compared to placebo, but patients with the higher levels of 0.9–1.1 ng/ml had a 2.6% increase, and those with ≥1.2 ng/ml exhibited an 11.8% increase during the 37-month follow-up. Since this was a *post hoc* analysis, repeat serum digoxin levels were not evaluated. Based upon this study, however, the authors concluded that levels ≥1.2 ng/ml may be harmful and may not provide any clinical benefit, whereas 0.5–0.8 ng/ml may be the optimal therapeutic range for men with heart failure and left ventricular dysfunction (7). In 2005, Adams and colleagues published their analysis of the relationship of the serum digoxin level and mortality among women, who had been enrolled in the DIG trial. They confirmed the same mortality risk among women who had serum levels ≥1.2 ng/ml at 1 month (47).

A 2015 meta-analysis by Chamaria et al. involving 12 studies and 321,944 patients, identified a 25% increased risk of mortality in patients with atrial fibrillation taking digoxin. If heart failure was also present, this increased mortality risk was not observed. The limitation of this meta-analysis was the lack of correlation with serum digoxin levels (48). The mortality risk associated with digoxin was again evaluated in a systematic review and meta-analysis of observational studies by Ziff et al. in 2015. They evaluated 52 studies involving 612,845 patients in which digoxin was prescribed for atrial fibrillation, heart failure, or both. Studies were of differing study design methods and included all observational and randomized controlled trials. When compared to a control group, “the pooled risk ratio for death with digoxin was 1.76 in unadjusted analyses (1.57 to 1.97), 1.61 in adjusted analyses (1.31–1.97), 1.18 in propensity matched studies (1.09 to 1.26) and 0.99 in randomized controlled trials (0.93 to 1.05)” (49). Additionally, “Limited information on digoxin doses suggests that lower serum digoxin concentrations of between 0.5 and 0.9 ng/ml were associated with improved prognosis, whereas higher concentrations correlated with increased mortality” (49). In 2015 another meta-analysis by Vamos et al. identified 19 reports, involving 326,426 patients, that dealt with digoxin associated all-cause mortality. The authors concluded: “This meta-analysis of the contemporary literature indicates that digoxin therapy, particularly without proper serum level control, is associated with an increased mortality risk in patients with atrial fibrillation and congestive heart failure” (50).

In 2016, a systematic review and meta-analysis by Qureshi et al. evaluated 9 randomized trials and 10 observational cohorts with digoxin use in atrial fibrillation. The authors identified a 27% increased risk of all-cause mortality among digoxin users (51). A limitation of this review is that one of the studies included in this analysis, Freeman et al., 2015, included 4,231 patients with atrial fibrillation which were “newly started” on digoxin to 10,556 patients with atrial fibrillation not on digoxin. The results of this study reported a 63% increase in hospitalization and 71% increase in death with digoxin use during the period of 1/1/2006–6/30/2009. The mean daily dose of digoxin did not vary between those who died and those who did not. However, they cannot exclude the possibility that an elevated serum digoxin level may have contributed since levels were not consistently measured. Mean serum digoxin levels were higher among those who died (1.151 ng/ml) compared to 0.935 ng/ml who survived. Unfortunately, these levels were not necessarily obtained immediately prior to death and based on arbitrary digoxin levels performed during the study period (52).

An observational study published in 2018, by Lopes et al. investigated whether digoxin use for atrial fibrillation was independently associated with mortality and whether the presence of heart failure was a confounder. The results demonstrated a linear correlation between an increased risk of death, the baseline digoxin level, and whether the patient was started on digoxin during the study timeframe (53). There was no increased risk if the baseline serum digoxin level was <0.9 ng/ml, but a 56% increased risk was identified if the digoxin level was ≥1.2 ng/ml. Additionally, a 4% higher risk of mortality was found with each 0.1 ng/ml increase in the baseline digoxin level. Sudden cardiac death occurred twice as often in new digoxin users compared to matched controls not on digoxin. They noted that patients already taking digoxin at the onset of the trial had a survival benefit by either demonstrating tolerance or having previously survived a potentially harmful event. A major limitation noted by the authors of this study was the lack of serum digoxin levels during the study period or after initiation of digoxin for atrial fibrillation. Their conclusions indicated that digoxin should be carefully monitored to maintain therapeutic levels since there was a direct increase in mortality with levels ≥1.2 ng/ml regardless of heart failure status.

The RATE-AF trial, published in 2020, demonstrated the effectiveness of low dose digoxin for atrial fibrillation (54). The randomized trial included 160 patients with chronic atrial fibrillation. The mean age of study participants was 76 years, 46% were female, and classified as New York Heart Association Class II or above. Participants were blinded to either digoxin or bisoprolol for 12 months. Digoxin levels were monitored at 6 months and as necessary during dosage adjustments to prevent supratherapeutic levels. The primary endpoint was patient-reported quality of life at 6 months and results did not demonstrate a significant difference between the two trial arms. Of the secondary end points evaluated there were 17 at 6 months of which 16/17 showed no significant difference 20 at 12 months with 8/20 favoring digoxin. There were 29 adverse events in the digoxin group compared to 142 in the β-blocker arm (*P* = 0.005). Digoxin was also associated with greater reductions in NYHA class and serum levels of the natriuretic peptide, NT-proBNP, at the end of the study. At 6 months, 73/76 patients were still on digoxin with a mean digoxin level of 0.78 ng/ml and mean dose of 161 mcg/day. Digoxin toxicity was not observed in this trial, likely due to the goal of maintaining a serum digoxin level below 0.9 ng/ml (54).

The risk of increased mortality from digoxin therapy for atrial fibrillation was also examined in a meta-analysis, by Wang and colleagues, in 2021. This analysis reviewed 29 studies representing 621,478 patients (55). They identified that digoxin therapy for atrial fibrillation resulted in a 17% increase in all-cause mortality regardless of whether congestive heart failure was present. Mechanisms proposed for the increased mortality included cardiotoxicity, exacerbation of platelet activation in patients with atrial fibrillation resulting in increased cardiovascular disease, digoxin's narrow therapeutic index, and potential for drug-drug interactions. Serum digoxin levels were not available in most of the studies analyzed resulting in a major limitation of this review. They concluded that “digoxin might be an additional choice for heart rate control in patients with both atrial fibrillation and heart failure, particularly in patients who are unable to tolerate β-blockers or do not achieve their target heart rate. The authors, however, went on to suggest that digoxin should be used cautiously with appropriate serum concentration monitoring to avoid digoxin toxicity”.

In 2022, Gerakaris et al. reviewed 15 observational studies evaluating the impact of digoxin on heart failure patients with atrial fibrillation. The authors noted that although it is unclear whether a higher risk of mortality from digoxin exists, low doses should be prescribed due to its narrow therapeutic index (40).

### 1.5. Formal recommendations for reduction of digoxin therapeutic levels

Since 2003, utilizing low doses of digoxin to achieve levels <1.0 ng/ml have been recommended by individual authors for heart failure (10, 56–58).

I. 2008 European Society of Cardiology (ESC) Guidelines for the Diagnosis and Treatment of Acute and Chronic Heart Failure 2008 recommendations for patients with heart failure stated: “The therapeutic serum concentration should be between 0.6 and 1.2 ng/ml, lower than previously recommended” (59).
II. 2013 American College of Cardiology Foundation (ACCF)/American Heart Association (AHA) Heart Failure Guideline further suggests that a digoxin plasma level should be 0.5–0.9 ng/ml if being used for heart failure and included a brief discussion about potential drug interactions (45). No update was made to this recommendation in the 2019 American Heart Association (AHA)/American College of Cardiology/Heart Rhythm Society (HRS) guidelines for atrial fibrillation management (60).
III. 2022 American Heart Association (AHA)/American College of Cardiology (ACC)/Heart Failure Society of America (HFSA) Guidelines for Management of Heart Failure: A Report of the American College of Cardiology/American Heart Association Joint Committee on Clinical Practice Guidelines suggested the upper therapeutic limit for digoxin for heart failure to be 1.0 ng/ml. A higher risk of death occurs with serum concentrations ≥1.2 with recommendations to use low dose digoxin (61).

Although clinical guidelines from the major cardiology thought leaders have translated scientific evidence into clinical practice for heart failure, there remains a lack of consensus on a definitive therapeutic range for digoxin when used for atrial fibrillation. The maximum target serum digoxin level for atrial fibrillation is now recommended by some to be <0.9 ng/ml (37) or ≤1.2 ng/ml (62). In 2016, Benlmouden and Billaud concluded in their manuscript *Evidence Based Digoxin Therapeutic Monitoring a Lower and Narrower Therapeutic Range* that “The target serum digoxin concentration should be 0.5–1.0 ng/ml” (63) while in 2018 Whayne supported “low doses” with monitoring to prevent supratherapeutic levels (27).

Several trusted medical sources continue to list the therapeutic level for digoxin up to 2.0 ng/ml including the FDA prescribing information monograph. The last revision by the FDA for digoxin monitoring was in 2016. It advises that a digoxin level less than 0.5 ng/ml may be inadequate and levels >2.0 ng/ml are associated with toxicity without increased benefit (24). Other examples of the disparate recommendations for digoxin therapeutic and toxic levels are noted in Table 6. While not all inclusive, the data clearly demonstrates the need for a consensus statement for digoxin's therapeutic range due to the vast disparity in the current recommendations.

**Table 6:** References published on the internet regarding therapeutic and toxic ranges for digoxin.

Source^ref^	Therapeutic range	Toxicity level	Additional comments	Last date this material was updated on the internet if identified.
BMJ Best Practice (64)	0.6–1.2 nmol/L (0.5–0.9 ng/ml)	>0.9 ng/ml	N/A	1/31/23
Clinical (65) Pharmacology & Toxicology Pearl of the week Digoxin Monitoring	0.6–1.2 nmol/L (0.6–0.9 ng/ml)	>2.6 ng/ml	Sources for this information: Canadian Cardiovascular Society and the American Academy of Family Physicians Written by the Calgary Clinical Pharmacology Service	1/24/2020
Epocrates.com (26)	0.5–0.9 ng/ml for HF^a^ 0.8–2.0 ng/ml for AF^a^	>2.0 ng/ml	Timing: just before next dose or >6 h after last dose Monitoring parameters: Cr, electrolytes, HR at baseline, then periodically; serum levels	11/23/18
LabTests online UK (66)	See discussion	See discussion	The appropriate (“target”) range for digoxin has been established over time as 0.5–2.0 mcg/L for patients being treated for heart failure. Several newer studies suggest a narrower range, 0.5–1.0 mcg/L, may be appropriate for some patients. The recommended range for patients with a/node/264 is 1.5–2.0 mcg/L. *NB*: µg/L = ng/ml	9/25/2018
Medscape (67)	0.8–2.0 ng/ml	>2.4 ng/ml	The toxic range for digoxin is greater than 2.5 ng/ml. About 10% of patients may show toxicities at levels less than 2 ng/ml (particularly in hypokalemia, hypomagnesemia, hypoxia, heart disease, and hypercalcemia.	11/21/2019
StatPearls (68) Digoxin chapter by David, MD and Shetty	0.5–2.0 ng/ml	≥2.0 ng/ml	N/A	9/5/2022
StatPearls (69) Digitalis Toxicity By Rehman, R, Dawson, A. H, Hai, O.	0.5–0.9 ng/ml in Toxicokinetics section 0.5–2 ng/ml in Evaluation section	Not discussed	Important to consider that concentration does not necessarily correlate with toxicity.	5/8/2022
UpToDate (18)	<1.0 ng/ml	>1.0 mg/ml	N/A	8/16/2022

^a^ AF, atrial fibrillation, HF, heart failure.

The recognition of a need to revise the current reference range for digoxin used by laboratories, occurred in the 2013. A Letter to the Editor of The Journal of the American Medical Association (JAMA) by Hauptman et al., shared the results of a survey on serum digoxin therapeutic ranges used by the top 100 hospitals rated by Thomson-Reuters (70). Of the 60 hospital laboratories that responded to the survey only two had the upper therapeutic limit of 1 ng/ml. Two others reported a range of 0.8–1.5 ng/ml, while the remaining 56 used 2.0–2.5 ng/ml as the upper limit for their digoxin therapeutic range.

In preparation for this manuscript, ten nationally recognized laboratories were contacted in 2023 by phone to determine their therapeutic ranges for digoxin, only 4/10 had updated the upper limit of the therapeutic range for digoxin to be 0.9–1.2 ng/ml, the remaining laboratories still referenced 0.8–1.0 ng/ml as the lowest accepted therapeutic level and 2.0–2.4 ng/ml as the upper level. If a value was lower than 0.8 or 1.0 ng/ml it was “flagged” as low.

### 1.6. Proposed digoxin monitoring recommendations

Scientific knowledge is in a constant state of flux. The endless stream of novel information requires periodic reevaluation of available evidence. Serum drug levels and monitoring are not exempt from this approach. It is for these reasons that the serum digoxin monitoring recommendations below are being proposed.

1. Therapeutic drug monitoring of serum digoxin should be regularly performed in the following situations:
  a) to confirm that the patient's level is in the therapeutic range
  b) when a provider suspects toxicity
  c) when renal function testing is indicated
  d) one week after starting a new medication.
    Serum electrolytes and magnesium should also be done at that time.
2. A serum digoxin level is strongly recommended 7–10 days after initiation of any new medication or if discharged from the hospital after being started on digoxin for the first time. The patient should be given an order for laboratory testing at the time of discharge.
3. Serum digoxin levels are not indicated after administration of a loading dose of digoxin for rate control in the hospital or ED and should be only checked after the patient has been on digoxin for 7–10 days.
4. Serum digoxin levels should not be obtained after the administration of Digoxin Immune Fab for acute digoxin toxicity since such administration will result in falsely elevated digoxin levels.
5. Serum digoxin levels should be obtained on admission to a hospital and subsequent levels should be done every 24–48 h to monitor for toxicity if new drugs are started.
6. Patients should be given a “standing order for a serum digoxin level for 6–12 months or as long as the laboratory policy allows” and educated on the necessity to have their serum digoxin level measured whenever they are started on a new medication by another provider (such as a dentist, podiatrist, urgent care, etc.). Education should also include the importance of a therapeutic level between 0.5 and <0.9 ng/ml.
7. All patients taking digoxin and their families should be educated regarding the signs and symptoms of digoxin toxicity. Patients presenting to an urgent care facility should be advised to inform providers that they are taking digoxin and request a serum digoxin level to be done to assure the level is not >0.9 ng/ml.
8. Patients must inform any provider when their last dose of digoxin was taken.
9. Patients should be encouraged to wear a medical alert bracelet identifying that they are taking digoxin.
10. Patients should be advised to take the digoxin in the evening to ensure that a level obtained the next day will be done at least 8 h after their dose.
11. Patients presenting to the ED or urgent care and are on digoxin should have a level taken if having any symptoms that might be related to digoxin toxicity. Careful consideration as to when the last dose of digoxin was taken is necessary for an accurate interpretation of the value.
12. Laboratory targets for digoxin need to be updated based on current clinical guidelines and laboratory results should reflect the following information for providers: “*The recommended therapeutic range for digoxin is: 0.5–0.9 ng/ml. There is an increased risk for complications/mortality > 0.9, and risk for toxicity if levels are > 2.0. Levels below 0.5 may be efficacious for some patients and clinical evaluation is warranted before dose is increased.”*
13. Before prescribing a new medication to a patient on digoxin, providers should perform a careful medication history and evaluate for drug-drug interactions. Note that many EHR systems, depending on the sensitivity elected within the EHR, may not include all of the potential drug-drug reactions that have been reported in the literature. This may limit a provider's ability to be alerted of a potential drug interaction with a moderate risk for toxicity. Therefore, we highly recommend that the patient be given an order for a repeat serum digoxin level 7–10 days after starting any new medication and advised provide education regarding potential symptoms of digoxin toxicity.

## 2. Conclusion

Current therapeutic drug monitoring guidelines for digoxin requires a major change in philosophy. The decades-old monitoring recommendations of obtaining digoxin levels if the patient is suspected of being toxic or to confirm compliance is no longer appropriate. A growing concern in our aging population is polypharmacy and an increasing risk of drug-drug interactions that include digoxin. Numerous meta-analyses have clearly identified that higher doses of digoxin are associated with increased mortality. Recommendations for a lower upper therapeutic range have been proposed in the cardiovascular literature since 2008 yet not fully adopted by the medical community. As with other narrow therapeutic index drugs, digoxin needs close, frequent monitoring to prevent toxicity and laboratories need to revise the therapeutic range to reflect efficacy and not toxicity.
