# Supplementary material for: Review: Failure of current digoxin monitoring for toxicity: new monitoring recommendations to maintain therapeutic levels for efficacy
Source: Front Cardiovasc Med. 2023 Jul 3;10:1179892. doi: 10.3389/fcvm.2023.1179892 (PMC10350506; doi:10.3389/fcvm.2023.1179892)
Supplement: Supplementary file 1 [file Table1.docx]

***Supplementary Material***

**Review: Failure of Current Digoxin Monitoring for Toxicity. New Monitoring Recommendations to Maintain Therapeutic Levels for Efficacy.**

**Sridhar Rao Gona, MS, MHA, BS. PHARM1*., Joel Rosenberg, MD, Ria C. Fyffe-Freil, PhD, Janet M. Kozakiewicz MS, PharmD, FASHP, Mary E. Money, MD, FACP**

*****Correspondence: Sridhar Rao Gona: [Sridhar.Gona@meritushealth.com](mailto:Sridhar.Gona@meritushealth.com)

**Case Report of Digoxin Toxicity**

The patient is an 80-year-old female who was started on digoxin 0.125 mg every other day in 2019 due to paroxysmal rapid atrial fibrillation/flutter that was not being controlled by metoprolol succinate 50 mg extended release (24-hour) tablet. Past medical history includes atrial fibrillation, sick sinus syndrome with status post dual chamber cardiac pacemaker placement, hypertension, chronic kidney disease, hyperlipidemia, diabetes, chronic back pain due to severe scoliosis, history of malignant melanoma, gastrointestinal reflux disease, renal calculi, congestive heart failure, mitral insufficiency, anxiety/depression, and hypothyroidism. Prior surgeries include unsuccessful atrial ablation, lumbar spinal fusion, pacemaker placement in 2015, appendectomy, cholecystectomy, hemicolectomy, hysterectomy, splenectomy, lithotripsy, and mitral valve repair transcatheter (MitraClip TEER) for severe mitral insufficiency on 2/18/21.

The patient’s digoxin was discontinued in 2019 due to chronic kidney disease and flecainide was initiated. Digoxin was restarted in June 2021 because of recurrence of rapid paroxysmal atrial fibrillation/flutter which was not controlled by metoprolol 50 mg bid, and flecainide 50 mg bid. A serum digoxin level 1 month after starting digoxin 0.125 mg daily was 0.23 ng/ml, and since her symptoms were not controlled the dose was increased to 0.125 alternating with 0.250 mg every other day. Repeat serum digoxin levels on 7/20/21and 8/2/21 were 0.66 ng/ml and 0.81 ng/ml, respectively. Other medications on board during this time included: acetaminophen, amlodipine, apixaban, B Complex-Biotin-FA, Vitamin D-3, Coenzyme Q10, colestipol, cyclobenzaprine, diclofenac gel, fenofibrate, fluoxetine, furosemide, glucosamine-chondroitin, levothyroxine, losartan-hydrochlorothiazide, magnesium gluconate, metoprolol tartrate, multiple vitamins, omeprazole, potassium citrate, tramadol, zolpidem, and the flecainide 50 mg bid.

The patient presented to the ED on 10/12/21 with symptoms of acute gastritis manifested by weakness and diarrhea. A repeat serum digoxin level was obtained on 10/12/21 at 12:36 pm, within 6 hours of the last dose, and was 1.13 ng/ml. No change was made to her digoxin regimen at that time, and she recovered within 48 hours and continued to do well without further difficulties.

In December 2021 the patient was started on Trulicity 0.75 mg/0.5 ml injection weekly for diabetes, after consultation with her nephrologist. The zolpidem was discontinued, and trazadone 50 mg was initiated. Over the next 12 weeks she lost 15 pounds and reported increased episodes of fatigue, abdominal pain with diarrhea and difficulty sleeping. Due to insomnia, the trazodone was increased to 100 mg. On 3/22/22 she was seen by her cardiologist and her weight had decreased another 6#. Her complaints now included lack of energy and depression noting “I can’t do anything”. No lab tests were ordered at that visit. She was seen on 4/22/22 by a new primary care provider and reported “I’m not again well, physically or mentally”. She also indicated at that visit “that she was more forgetful”. Her weight had further decreased to 162#, representing a 23-pound weight loss since 10/12/22, prior to starting Trulicity.

The patient presented to the Meritus Medical Center Emergency ED on 7/10/22 complaining of fatigue, arm pain, and rash due to an insect bite on her right thigh. She was diagnosed with cellulitis on the thigh (although a description of the rash was not included in the ED note) and started on doxycycline hyclate 100 mg bid for 7 days. No drug-drug reactions were triggered, in the electronic health record (EHR), when the order for doxycycline was electronically placed. During this ED visit her weight was 158#. Labs results drawn in the ED included: Sodium 135, Potassium 3.3, BUN 24, and Creatinine 1.49, as well as mild elevations of liver enzymes. No digoxin level was obtained. A Lyme Disease test was drawn to rule out Lyme disease, which was later confirmed, and doxycycline was continued until 7/28/22 by her primary care provider (see below).

On 7/12/22 the patient saw another primary care provider in the same office, who recorded that “the patient indicated activities such as getting out of bed/taking a shower cause extreme fatigue which is new in the past week”. The (EHR) notes from that visit stated that “she still had diminished appetite and had some episodes of vomiting that she thought might be due to Tylenol although she had been taking a low dose”. She has not been eating due to nausea which preceded the ED evaluation and initiation of doxycycline. On 7/28/22 the patient was seen by her principle primary care physician for nausea and vomiting which was thought to be due to the doxycycline and was subsequently discontinued. Her liver enzyme results on 7/19/22 had returned to normal and the nausea could not be explained. On 8/2/22 the patient was seen by an infectious disease physician because of her symptoms and concerns by the patient that it might associated with the Lyme disease. Her weight at that office visit was 152# and her primary complaint was “malaise/fatigue.” A limited abdominal ultrasound was done on the same date that was unremarkable, demonstrating decreased hepatic steatosis compared to a prior ultrasound findings and cholecystectomy. The patient was reassured that her symptoms were not due to Lyme Disease and no additional orders were placed. One day later on 8/3/22, her husband called her primary care physician again since she was now having trouble keeping any liquids down and had a worsening of her vomiting. The staff taking the call recorded: “He was suspicious that this was now due to the Trulicity since it was worse after she took her shot last night.” The primary care physician had not had a chance to return the call by the time her husband contacted another provider on 8/4/22 for a second opinion. After a telemedicine consultation and close review of the patient’s chart, laboratory studies were ordered immediately for 8/5/22 with instructions for the patient NOT to take her digoxin before getting the lab done. Her serum digoxin level at 10:45 am (at least 24 hours after her last dose on 8/4/22) was 3.31.

Digoxin was immediately stopped, and the patient noticed rapid resolution of her symptoms. Digoxin was not restarted for a week and a repeat serum level (0.68) was obtained on 8/24/22 after the patient had resumed Trulicity for 2 weeks. Her digoxin was restated at a lower dose of 0.125 mg every other day. The patient’s nausea completely resolved and she was able to resume light gardening and housework. Her symptoms of confusion resolved as well as apathy and inability to get out of bed. She reported that her episodes of atrial fibrillation and flutter were infrequent and short in duration.

Laboratory results as of 2/7/2023: serum digoxin level 0.56 ng/ml, BUN 34, Creatinine 1.48, GFR 36.

Based on the history reported to the primary care provider, the digoxin toxicity was likely due initially to the Trulicity, and then further exacerbated by the doxycycline.
